# Moving from tangibility toward digitalization: investigating team dynamics and facilitator support among medical students in conventional and digital small-group tutorials

**DOI:** 10.1186/s12909-022-03893-8

**Published:** 2022-11-28

**Authors:** Chia-Ter Chao, Yen-Lin Chiu, Chiao-Ling Tsai, Mong-Wei Lin, Chih-Wei Yang, Chao-Chi Ho, Yen-Yuan Chen, Chiun Hsu, Huey-Ling Chen

**Affiliations:** 1 Nephrology Division, Department of Internal Medicine, National Taiwan University Hospital, Taipei, Taiwan; 2 Center of Faculty Development and Curriculum Integration, National Taiwan University College of Medicine, Taipei, Taiwan; 3 Graduate Institute of Toxicology, National Taiwan University College of Medicine, Taipei, Taiwan; 4 Graduate Institute of Medical Education and Bioethics, National Taiwan University College of Medicine, Taipei, Taiwan; 5 Department of Radiation Oncology, National Taiwan University Hospital, Taipei, Taiwan; 6 Department of Surgery, National Taiwan University Hospital, Taipei, Taiwan; 7 Department of Medical Education, National Taiwan University Hospital, Taipei, Taiwan; 8 Department of Emergency Medicine, National Taiwan University Hospital, Taipei, Taiwan; 9 Chest Medicine Division, Department of Internal Medicine, National Taiwan University Hospital, Taipei, Taiwan; 10 Department of Medical Oncology and Department of Medical Research & Education, National Taiwan University Hospital Cancer Center, Taipei, Taiwan; 11 Department of Pediatrics, National Taiwan University Hospital, Taipei, Taiwan

**Keywords:** Collaborative learning, Facilitator support, Medical education, Online education, Small-group tutorial, Team dynamics

## Abstract

**Background:**

Small group tutorials (SGT) promotes self-directed learning and is widely used in medical education. The coronavirus pandemic (COVID-19) has accelerated the trend toward SGT digitalization, with unclear effect. We hypothesize that team dynamics and facilitator support influence SGT satisfaction in digital versus conventional SGT.

**Methods:**

During the spring semester of year 2021, medical students (the second, third, and fourth year; *n* = 433) participating in conventional face-to-face and digital SGT curricula were enrolled. Participating students completed the collaborative learning attitude scale (including team dynamics, team acquaintance, and facilitator support dimensions) and teamwork satisfaction scale, previously validated for small-group collaborative learning, and chose preference between conventional or digital SGT in future curricula. Exploratory factor analysis (EFA) was performed to extract the essential structural factors of these scales. Paired t-tests were conducted to compare differences in different dimensions and satisfaction between the conventional and digital SGT settings. Two sets of multiple regression analyses were done; one with team satisfaction scale results and the other with preference for digital SGT as the dependent variable were used to evaluate determinants of these two variables.

**Results:**

The EFA results revealed that the original collaborative learning attitude scale was concentrated on two dimensions: team dynamics and facilitator support. No significant differences were noted between the SGT settings for the two dimensions and teamwork satisfaction. Regression analyses showed that teamwork dynamics was independently correlated with teamwork satisfaction in both conventional and digital SGT. Facilitator support was positively correlated with teamwork satisfaction in conventional, but not digital SGT. Higher teamwork satisfaction was an important determinant of preference for digital SGT among medical students.

**Conclusions:**

Team dynamics were closely linked to teamwork satisfaction among medical students in both conventional and digital SGT, while the role of facilitator support became less obvious during digital SGT.

## Introduction

Collaborative learning (CL), a widely adopted pedagogical method, promotes students’ learning experiences through fostering their teamwork skills. Educational approaches engrained with more active, social and engaging elements tend to encourage deep learning and high-order thinking. Among the spectrum of CL, problem-based learning, small group tutorial (SGT), team-based learning, etc. each has its own features. SGT also represents an important advancement in education strategy during recent decades [1]. As a vibrant approach aiming to promote learner-centrality, SGT helps participants cultivate communication abilities [2, 3]. SGT has been shown to be a relatively effective and preferable method for medical students complementary to traditional didactic lectures. Within the SGT curricula, learners are guided along the problem-solving path through critical thinking facilitated by a skillful tutor [4]. This format differs considerably from the traditional didactic lectures, through allowing participants to immerse themselves in complex discussions, skill practicing, and reflect on their own performances [5].

National Taiwan University College of Medicine (NTU-CM) has adopted SGTs in medical education for more than 20 years. Through a re-design of curricular program, cross-generational staff in NTU-CM created integrated courses spanning from biological and clinical sciences to medical humanity, medical ethics, community medicine and social sciences using SGTs with cases [6]. Each year we recruited board-certified physicians from affiliated hospitals to serve as SGT tutors. Most of them were considered non-expert regarding specific subject matter (e.g., cardiovascular pharmacology). We emphasized the concept of ‘teachers/ tutors as learners’ and the CL in our program involves students and also tutors. We incorporated SGT and didactic lectures into an introductory course of clinical medicine, categorized by organ systems. This approach allowed more active roles of the facilitators in the SGT curriculum. During the SGT, cases in the syllabus were initially translated from English materials in the American context (New Pathway program within the format of problem-based learning) [7], but later were adapted with cultural context-appropriate question-oriented learning [8]. Students were further empowered by facilitators to transcend fixed discussion outlines and to leverage their motivation for developing newer questions and for self-directed answer discovery [8]. An inductive and introspective instead of directed type teaching [9] was harnessed to consolidate student motivation. Questions in each case can be tutor-designed or learner-generated, depending on discussion content [6, 8]. The SGT in NTU-CM aims to promote the internalization of life-long learning among 2^nd^ to 4^th^ grade medical students and recently to the students from the school of pharmacy, nursing, and medical laboratory technology. From this perspective, the SGT in NTU-CM is evolving and adapts itself continuously to culture-sensitive context while still emphasizes individual learning and group harmonization [8].

Facilitators and participating students are the core members of a typical SGT curriculum, the success of which hinges on teaching techniques, student motivation, and team dynamics [10]. A recent report dictates that interactions, discussions, and collaborations between learners serve as the cornerstone of a successful SGT [11]. However, this premise has been challenged by the sweeping tide of the coronavirus pandemic (coronavirus disease-2019; COVID-19). Social distancing and school/city lockdown have negated the possibility of conventional face-to-face SGT curricula. Facilitators and students are also tasked with the requirement to meet virtually. Despite such necessity, this trend pops the question on how the role and the interaction between members likely change during online SGTs, and how medical students feel during their collaborations online.

In NTU-CM, we previously surveyed medical students during the initial phase of transition from conventional to digital SGTs. Based on their responses, we demonstrated that team dynamics and facilitator features played an important role in affecting learning confidence [12]. A prior study showed that during SGT, such learning confidence can be affected by students’ perception of teamwork satisfaction [13]. This inspires us to hypothesize that team dynamics and facilitator support play a major role in determining teamwork satisfaction among SGT participants across in-person and virtual settings. Indeed, better student engagement and interactions promoted students’ satisfaction during online collaborative learning [14]. Better team dynamics contributed to more enjoyable team atmosphere, greater job satisfaction, and a greater likelihood of optimized clinical performance [15]. Team effectiveness and work satisfaction could be positively influenced by better team dynamics [16]. We specifically focused on the influences of team dynamics, because team dynamics embedded in collaborative learning have been proposed to influence learning efficacy by promoting psychological safety among peers [17]. Optimized team dynamics are considered vital elements of learning strategies such as team-based learning, and positively prepare healthcare professionals for collaborative work during their subsequent careers [18]. In addition, to make results comparable, we examine the contribution of team dynamics and facilitator factors to teamwork satisfaction in both conventional and digital SGT settings. It is shown that greater teamwork satisfaction is often accompanied by a positive attitude toward working with peers and potentially leads to better academic performance [19]. Teamwork satisfaction might correlate positively with team performance in collaborative online learning [20]. Therefore, we expected that determining how to enhance teamwork satisfaction could be translated into better student performance during our digital SGT curriculum.

## Materials and methods

### Study procedures

**Fig. 1:**
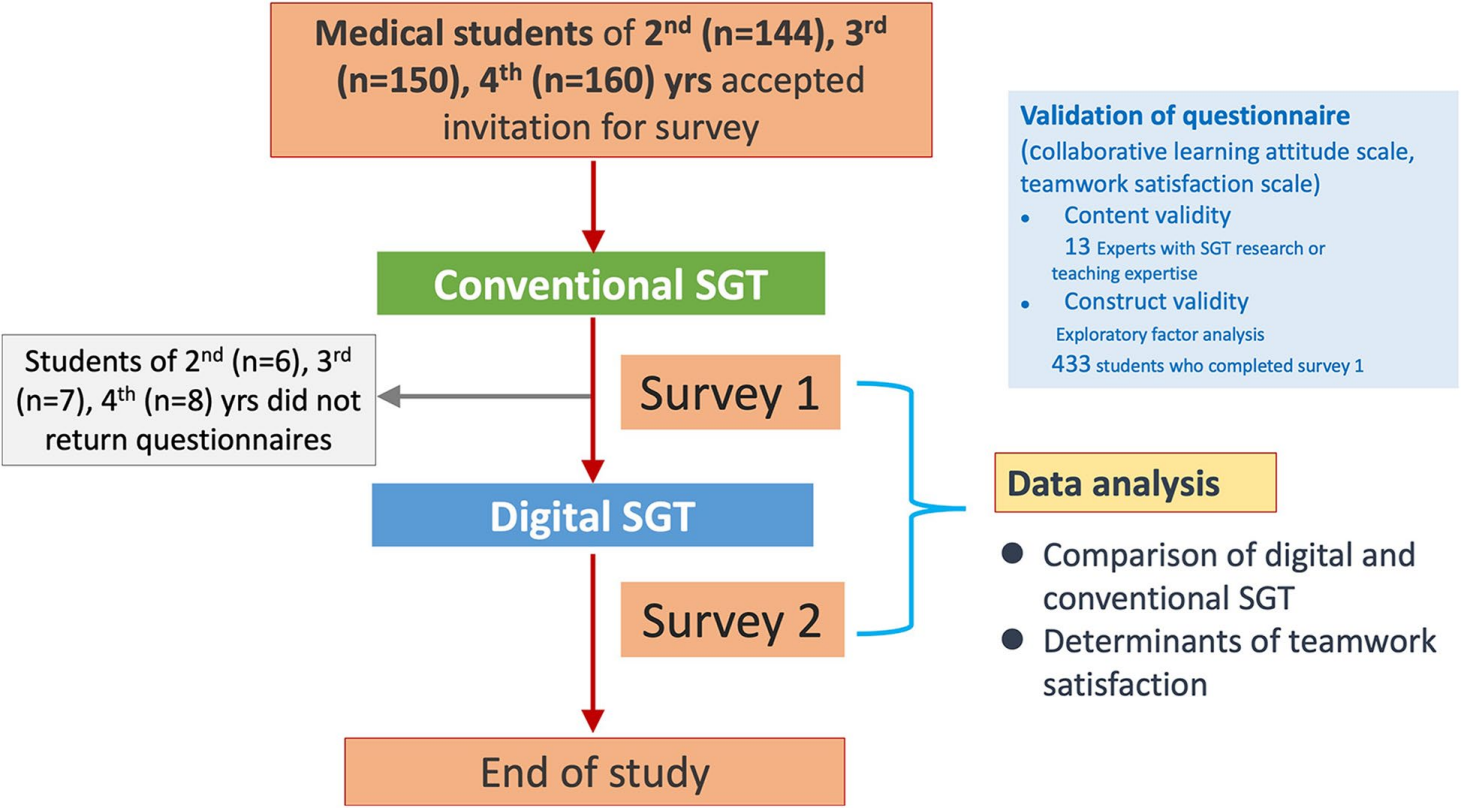
The procedure of the current study. *SGT, small group tutorial*

The setting of SGT curricula in NTU-CM remained face-to-face (i.e., “conventional SGT”) until early 2020, when the COVID-19 pandemic spread around the world and prompted the shift from conventional face-to-face SGT to a fully online learning platform (i.e., “digital SGT”) at NTU-CM since April 2020. Details of our digital SGT curricula has been described before [12, 21]. During the ensuing months in 2020, we developed a structured digital SGT curriculum based on input from the medical school educational committee meetings, medical education experts, experiences and feedbacks from teachers and students in the initial digital sessions [21]. Participants were asked to respond initially to two validated questionnaires to assess their learning attitudes, including team dynamics, and teamwork satisfaction after participation in the conventional SGTs. They were subsequently switched to digital SGT, and the same set of questionnaires were distributed thereafter. A brief outline of the study procedure is shown in Fig. 1.

Participant responses were recorded in an anonymized fashion based on a computed-generated de-identified method (i.e., the National Taiwan University Hospital (NTUH) Redcap system). Personal data recorded in this study included only the academic years of medical students and did not identify any other demographic features.

### Study participants

The mainstream medical education system in Taiwan consists of six years of undergraduate training and two years of postgraduate training. Conventional SGT curricula have been implemented at the NTU-CM for more than 20 years, involving subjects including medical humanities (second year), anatomy and physiology (third year), pathology and pharmacology (fourth year) [8]. The SGT curricula include designed course contents for each session, and involve physicians as facilitator aiming at integrated education of basic/clinical science as well as medical humanities. Medical students are divided into small groups of up to 10 students per group (mostly 6 to 8). Clinical cases and scenarios are incorporated into the outline of each session to facilitate learning and discussion. Physicians or surgeons from different disciplines are assigned to each small group as facilitators. For each semester, the SGT curricula are offered once weekly with 2-h of session length. A more comprehensive introduction to the SGT curricular content in NTU-CM has been reported in the literature [12, 22].

To continue optimizing course digitalization in response to the fluctuating COVID-19 pandemic [12], and also consider incorporating digital SGT into the course on a regular basis, we examined how team dynamics and facilitator factors influenced medical students’ perception in the form of teamwork satisfaction under different SGT settings. We invited all medical students of the second, third, and fourth year in NTU-CM to participate in this study during the fall semester of 2020 (Fig. 1).

### Study instruments

We chose questionnaires previously validated for application in online small-group collaborative learning [23], particularly insofar as digital SGT was a core and contemporary element of this study. Two instruments were employed. The first instrument comprises 20 items and assesses the overall student attitudes toward collaborative learning [23]. The instrument is divided into three dimensions: team dynamics, team acquaintance, and facilitator support. The second instrument used in this study is the teamwork satisfaction scale (10 items), which we used to measure students’ perception of their collaborative learning process during conventional and digital SGT curricula [23]. Items from both instruments were formatted in a five-point Likert-type scale. Each item was translated into traditional Chinese by one of the co-authors (CTC) and then back-translated into English for comparison. The original, translated, and back-translated versions of the two instruments were circulated to an expert advisory committee for their feedback. The committee members included researchers with expertise in undergraduate medical education, senior facilitators and coordinators of the SGT curricula, and facilitator/researchers from the Institute of Medical Education and Bioethics at NTU-CM. The translated instruments were then revised in an itemized fashion using consensus-reaching processes and iterative responses. All items were carefully reviewed for wording, phrasing, appropriateness, specificity/refinedness, and clarity, with redundant descriptions removed and ambiguous words recalibrated. As the existing literature rarely addressed team dynamic assessment among participants of conventional SGT, we chose to administer these instruments to our students participating in conventional and digital SGT, with phrasings specifically adapted to the respective scenarios based on expert consensus and text optimization.

We further ensured the validity of the two adapted instruments, that is, the extent to which the tool assessed the intended messages based on the content validity index. Content validity captures the degree to which each item matches a designated dimension. We used an empirical method to calculate the content validity index (CVI), a well-established tool for instrument development, and obtained the item-CVI (I-CVI) and scale-level CVI (S-CVI). Results from I-CVI ranged between 0 and 1, and we revised items for those with I-CVI between 0.7 and 0.79 while discarding items with an I-CVI below 0.7 [24]. We also calculated the average S-CVI (S-CVI/Ave) by summing the I-CVI of all items divided by the item numbers; an S-CVI/Ave ≥ 0.9 indicated excellent content validity for the instrument [24].

In this study, we first designed the study flow and evaluated questionnaire validity. We surveyed the students using the questionnaires after conventional and digital SGT. Data collection then followed. Facilitators in the conventional and digital SGT curricula were the same throughout the study period.

### Analytic approaches

We first used exploratory factor analysis (EFA) to extract the essential structural factors inherent in the original collaborative learning attitude scale applicable to our participants, following the principles outlined earlier [25]. After ensuring the content validity of the adapted instruments (collaborative learning attitude scale and teamwork satisfaction scale), we collected results from these instruments to all participating medical students after conventional and after digital SGT curricula. Students were later asked to choose preference between conventional or digital SGT. Data from conventional and digital SGT participants were linked using de-identified methods and curated for data integrity. The results generated from the validated instruments after EFA trimming were compared between participants of the conventional and digital SGT curricula using the paired *t*-test. Subsequently we used multiple linear regression analyses, with teamwork satisfaction scale results serving as the dependent variables, incorporating medical students’ academic year and results from different dimensions of the collaborative learning attitude scale. In addition, logistic regression analysis investigating factors associated with preference for future digital SGT curricula was performed, and incorporated medical students’ academic year, teamwork satisfaction, team dynamics, and facilitator support as variables. Statistical significance was set at *p* < 0.05, and all analyses were conducted using the SPSS (Statistical Package for the Social Sciences version 20.0).

### Ethical statement

The study procedure conformed to the ethical standards of the NTUH. The institutional review board of the NTUH has approved the protocol of the current study (approval NO. 202201024RIND). The institutional review board of the NTUH waived the need for informed consent, since this study was observational in nature and participant/data were already de-identified upon data collection. All methods used in this study were carried out in accordance with the Declaration of Helsinki. The project plan has been reviewed and approved in the University’s educational development unit, and is part of the continuous effort to improve medical education.

## Results

Thirteen experts were invited to rate the teamwork satisfaction scale and collaborative learning attitude scale to obtain the CVI. The I-CVI for the 10-item teamwork satisfaction scale ranged between 0.8 and 1.0, with a S-CVI/Ave of 0.967, suggesting excellent content validity. The I-CVI for the 20-item collaborative learning attitude scale was mostly above 0.8, except for two items (Nos. 5 and 19), with an I-CVI of 0.733. The two items were then subjected to expert consensus-based rephrasing to increase specificity and clarity. The S-CVI/Ave score of the revised Collaborative Learning Attitude Scale was 0.92.

A total of 454 medical students, including second year, *n* = 144 (31.7%); third year, *n* = 150 (33.0%); and fourth year, *n* = 160 (35.2%) students, were invited to participate in this study. Among them, 6 (4.2%), 7 (4.7%), and 8 (5.3%) second, third, and fourth year students, respectively, did not return questionnaires for the conventional SGT sessions, and were therefore excluded from analysis. Complete responses were obtained from all the remaining participants after the digital SGT session (Fig. 1).

After conducting EFA using responses from the conventional curricula, we found that the original three dimensions of the collaborative learning attitude scale—namely, team dynamics, team acquaintance, and facilitator support—could be concentrated to form two dimensions: team dynamics and facilitator support, containing 16 and 3 items, respectively (Table 1). One item, “*My team members communicate in a courteous tone*,” exhibited prominent cross-loading and was subsequently removed. The validated/recalibrated collaborative learning attitude contained 19 items, with a Kaiser–Meyer–Olkin (KMO) value of 0.971 and significant Bartlett’s test for sphericity (*p* < 0.001; Table 1), suggesting sampling adequacy for factorial analysis. The Bartlett’s test for sphericity showed that our data exhibited a good cohesion (*p* < 0.001; Table 1). For the EFA results of teamwork satisfaction scale, 10 items were grouped into one dimension, with a KMO value of 0.947 and significant Bartlett’s test for sphericity (*p* < 0.001; Table 2). In addition, the Cronbach’s α values for team dynamics, facilitator support and team satisfaction were 0.969, 0.815, and 0.954, respectively, showing satisfactory internal consistency. The EFA results and Cronbach’s α values remained essentially the same regardless of using conventional or digital SGT curriculum survey.

**Table 1:** Results of factor analyses from the original collaborative learning attitude scale (reference [23])

*Item NO*	*Item description*	*Team dynamics*	*Facilitator support*
13	My team members learn how other members wish to be treated and then act accordingly	0.841	
9	My team members communicate with each other frequently	0.830	
20	My team members clearly know their role during their collaboration	0.815	
16	My team trusts each other and works toward the same goal	0.793	
6	My team members share personal information to know each other better	0.782	
11	Communicating with team members regularly helps me to understand the team project better	0.775	
17	My team develops clear collaborative patterns to increase team learning efficiency	0.774	
12	My team members encourage open communication with each other	0.766	
8	Getting to know one another in my team allows me to interact with teammates more efficiently	0.762	
14	My team members provide all responses in a timely manner	0.757	
18	My team sets clear goals and establishes working norm	0.739	
19	My team has an efficient way to track the edition of documents	0.738	
5	My team members share culture information to know each other better	0.717	
4	My team is receiving feedback from each other	0.697	
7	My team members share their professional expertise	0.683	
15	I trust each team member can complete his/her work on time	0.670	
2	The instructor acts as a referee when our members cannot seem to resolve differences		0.852
1	My team is receiving guidance of the group project from the instructor		0.825
3	The support from the instructor helps my team to reduce anxiety among team members		0.678
KMO value		0.971	
Bartlett’s test		X^2^ = 7625.024 (*p* < 0.001)	
Total variance explained		70.126%	

*KMO* Kaiser–Meyer–Olkin test

**Table 2:** Results of factor analyses from the teamwork satisfaction scale (reference [18])

*Item NO*	*Item description*	*Load*
2	I like solving problems with my teammates in group projects	0.891
6	I enjoy the experience of collaborative learning with my teammates	0.883
5	I have benefited from my teammates’ feedback	0.883
4	I have benefited from interacted with my teammates	0.880
1	I like working in a collaborative group with my teammates	0.875
3	Interacting with the other members can increase my motivation to learn	0.854
7	Online teamwork promotes creativity	0.827
10	I gain online collaboration skills from the teamwork processes	0.818
8	Working with my team helps me produce better project quality than working individually	0.810
9	My team members are sharing knowledge during the teamwork processes	0.699
KMO value		0.947
Bartlett’s test		X^2^ = 3988.013 (*p* < 0.001)
Total variance explained		70.126%

*KMO* Kaiser–Meyer–Olkin test

### Comparison of student responses to conventional and digital SGT curricula

We subsequently examined whether the SGT setting affected students’ attitude toward and satisfaction with the curricula, which required collaborative learning in a small-group fashion (Table 3). No significant differences existed between the different SGT settings with respect to results pertaining to team dynamics and facilitator support dimensions (both *p* > 0.05). Moreover, perceived teamwork satisfaction among medical student participants was not altered by the implementation of the digital SGT curriculum (Table 3).

**Table 3:** Comparisons of teamwork satisfaction, instructor support, and team dynamics results between conventional and digital SGT curricula

*Variables*	Conventional SGT		Digital SGT		*t*	*P value*
	*Mean*	*SD*	*Mean*	*SD*		
Teamwork satisfaction	4.205	0.677	4.153	0.715	1.675	0.095
Instructor support	4.380	0.585	4.339	0.647	1.463	0.144
Team dynamics	4.214	0.623	4.254	0.672	-1.462	0.145

Note: paired t-tests, *SD* Standard deviation*, SGT* Small group tutorial

### Independent determinants of teamwork satisfaction among medical students as SGT participants

We investigated the determinants of teamwork satisfaction among medical students participating in conventional or digital SGT, including students’ academic year, facilitator support, and team dynamics results (Tables 4 and 5). Regression analyses revealed that among conventional SGT participants, better facilitator support (*p* < 0.001) and team dynamics (*p* < 0.001) were significantly associated with a greater degree of teamwork satisfaction, whereas students’ academic year was not (Table 4). Interestingly, among digital SGT participants, only better team dynamics (*p* < 0.001) were significantly associated with a greater degree of teamwork satisfaction, while better facilitator support and students’ academic year were not (Table 5).

**Table 4:** Results of regression analysis on teamwork satisfaction of conventional SGTs

*Variables*	*B*	*Standard error*	*β*	*t*	*P value*	*VIF*
Constant	0.074	0.133		0.553	0.581	
Grade 3^rd^ vs. 2^nd^	0.059	0.042	0.041	1.406	0.160	0.059
Grade 4^th^ vs. 2^nd^	-0.035	0.041	-0.025	-0.864	0.388	-0.035
Instructor support	0.150	0.041	0.132	3.677	< *0.001*	0.150
Team dynamics	0.823	0.039	0.758	21.082	< *0.001*	0.823

Note: *SG* Small group tutorial, *VIF* Variance inflating factor; adjusted R^2^ = 0.734; F = 299.722 (*p* < 0.001)

**Table 5:** Results of regression analysis on teamwork satisfaction of digital SGTs

*Variables*	*B*	*Standard error*	*β*	*t*	*P value*	*VIF*
Constant	0.058	0.118		0.493	0.622	
Grade 3^rd^ vs. 2^nd^	-0.041	0.040	-0.028	-1.036	0.301	1.390
Grade 4^th^ vs. 2^nd^	-0.010	0.040	-0.007	-0.259	0.796	1.377
Instructor support	0.037	0.040	0.033	0.909	0.364	2.085
Team dynamics	0.929	0.040	0.873	23.520	< *0.001*	2.106

Note: *SGT* Small group tutorial, *VIF* Variance inflating factor; adjusted R^2^ = 0.814; F = 417.604 (*p* < 0.001)

### Independent determinants of preference for digital SGT among medical students

Logistic regression analyses were further conducted to identify the factors affecting digital SGT preferences among the SGT participants (Table 6). We found that a higher teamwork satisfaction rating (*p* = 0.011) was an important determinant of the preference for digital SGT among all participants (Table 6).

**Table 6:** Results of logistic regression analysis on preference for digital SGT in the future

*Variables*	*B*	*Standard error*	*OR*	*95% CI*	*P value*
**Grade**					
Grade 3^rd^ vs. 2^nd^	0.358	0.295	1.430	0.803 – 2.549	*0.225*
Grade 4^th^ vs. 2^nd^	0.580	0.288	1.785	1.016 – 3.138	*0.044*
**Teamwork satisfaction (during digital SGT)**	1.065	0.419	2.902	1.277 – 6.594	*0.011*
**Instructor support**	-0.056	0.296	0.946	0.529 – 1.690	*0.850*
**Team dynamics**	-0.825	0.475	0.438	0.173 – 1.112	*0.082*

*CI* Confidence interval, *OR* Odds ratio, *SGT* Small group tutorial

**Fig. 2:**
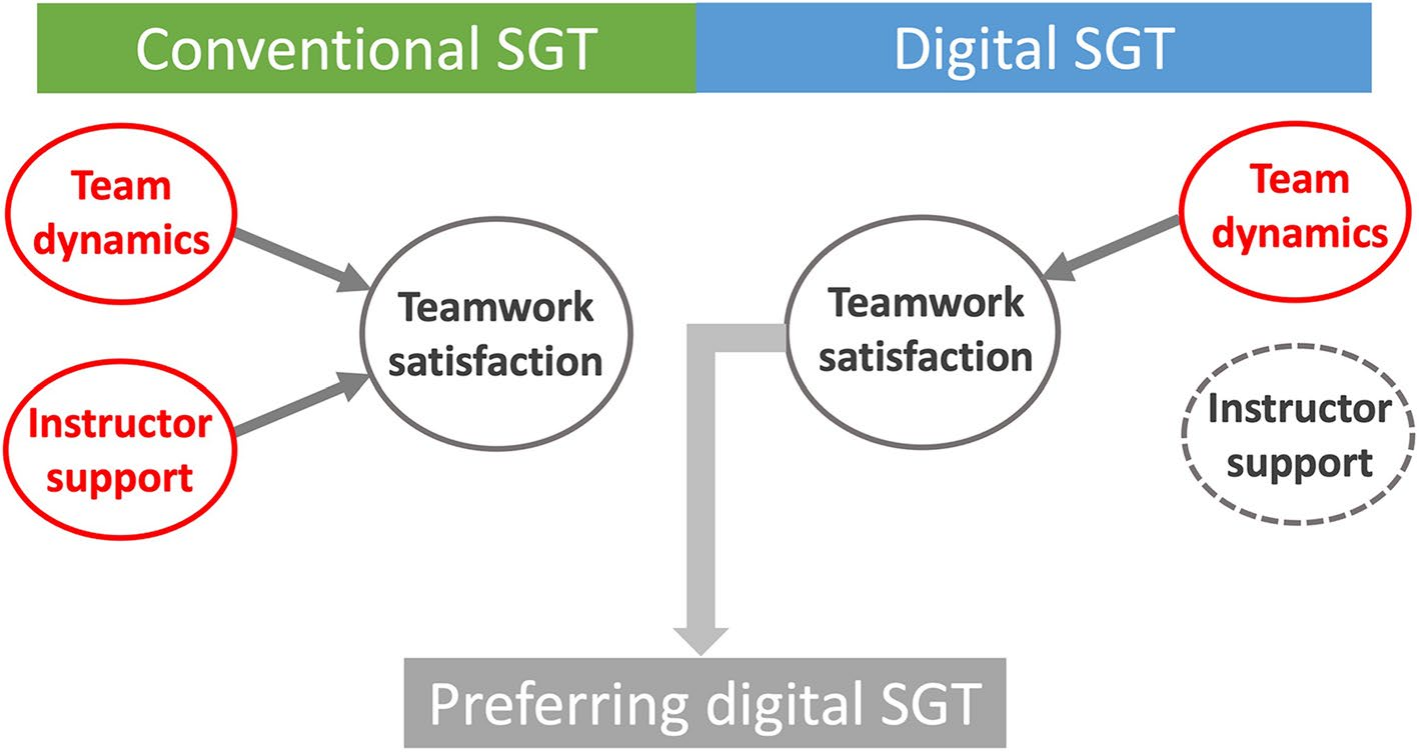
A summary diagram of our study findings. The dashed circle indicates the absence of associations. *SGT, small group tutorial*

A summary illustration is provided in Fig. 2.

## Discussion

In this study, we first validated existing instruments for assessment of interaction/satisfaction during the small-group collaborative learning process in our conventional and digital SGT course. We achieved adequate content validity among our participating medical students, with an S-CVI/Ave of 0.92. We found that no differences in self-rated team dynamics, facilitator support or teamwork satisfaction between conventional versus digital SGT. Interestingly, facilitator support was positively correlated with teamwork satisfaction in conventional, but not digital SGT. Teamwork dynamics was independently correlated with teamwork satisfaction across conventional and digital SGT settings. Regression analysis revealed higher teamwork satisfaction as an important determinant of preference for digital SGT among medical students (Fig. 2).

Several features differentiated our SGT curriculum from the conventional problem-based learning. Firstly, the collaborative learning in our program emphasized activities between the tutors (‘teachers/ tutors as learners’) and their students. Secondly, the program designers and tutors in our program played more active roles in introducing the ‘paradigms’, according to Thomas Kuhn [26], of clinical medicine to the students by constructing questions using their clinical expertise.

Teamwork efficiency and interaction status reportedly play an important role in small-group learning processes such as SGT. In this study, we intended to capture the landscape of intra-team interactions during SGTs of different settings using existing instruments, but researchers have opined that the use of a static assessment of team states may not fully capture the inherently dynamic interactions within healthcare teamwork [27]. Nonetheless, a comprehensive evaluation of all events and time-based observation can be laborious and time-consuming; moreover, our prior experiences indicated that off-table communication was common during digital SGT curricula [21], rendering the online monitoring of intra-SGT interaction less practical. Therefore, we believe that the questionnaire we used provide a glimpse of how SGT participants perceived about their team dynamics during curricula. Team dynamics include several important categories that benefit learning perceptions and work skills, such as interpersonal team processes and task-oriented effort [18]. The current study serves the purpose of providing useful tools for assessment of team dynamics. These tools can be of great value to further investigate vital contributors to collaborating outcomes among SGT participants.

Interestingly, we showed that teamwork dynamics independently correlated with teamwork satisfaction, an association consistently shown across different SGT settings (Tables 4 and 5). A prior study indicated that in online SGT, team dynamics were a potentially vital element for providing students with a sense of interdependence and mutual collaboration [28]. Better practices in team dynamics enhances student engagement with online course content and potentially promotes satisfaction [28]. This is in line with theories pertaining to online education indicating that learner motivation, curricular design, shared community building, and reflective practices are essential for maximizing learners’ gain [29].

On the other hand, an issue that deserves attention is the role of facilitator in online education, especially during small group learning and discussions. We found that the role of SGT facilitators appeared rather ambiguous and likely waned in a student-centric environment (Table 5). There are calls to uphold identity changes or the repositioning of facilitators as “co-participants” to better enhance learning experiences and efficacy during online teaching [30]. A recent study further found that the most influential factor in determining satisfaction with online courses among medical students was course design instead of facilitator issues [31]. Undergraduate medical students are found to value e-learning-based SGTs less than in-person SGTs, although the former still plays an adjunct role in learning promotion [32]. In addition, the role assumed by facilitators in SGTs is frequently to streamline discussions and to inspire self-directed learning [33], but this role can be worn during the transition from in-person to the digital environment due to restraint from time spent on atmosphere building, agenda/task re-focusing, and engagement issues [11]. These arguments may in part be responsible for the diminished association between facilitator support and teamwork satisfaction during digital SGT. Despite our observations, teamwork satisfaction cannot be treated as being synonymous with the overall learning outcomes. We believe that facilitators can still play an important role in orchestrating an optimal digital SGT and ensure the achievement of learning objectives. Our data may be partially explained by the fact that less-experienced facilitators were unfamiliar with a digital platform or the appropriate workflow of a digital SGT, provided less feedback to participants, thereby obscuring their images in the curriculum. On the other hand, experienced facilitators may be more versed in accommodating themselves swiftly to the digital platform. This issue should be taken into consideration during blended learning incorporating in-person and digital SGT for medical students.

Based on our findings, we propose several strategies to improve teamwork satisfaction in digital SGTs for undergraduate medical students. First, the learning curves of students and facilitators for digital curricula need to be strengthened. While the COVID-19 pandemic accelerates the transformation of education pedagogy, different generations of facilitators adapt at different speeds. Suboptimal performance from the facilitator side attenuates teamwork satisfaction, potentially reducing the extent of student engagement in curricular content and group processes. We previously found that repetitive practices of digital SGTs improved students’ confidence [12], and similar influence is expected for facilitators participating continuously in such curriculum. There are also feedbacks from students recommending that facilitators join a pre-course workshop on skill honing [21]. In addition, facilitators can have their mindset renovated, through being “creative”, upgrading one’s familiar teaching methods, and introducing subgroup exercises, as outlined by a recent opinion pieces [11]. In order to enhance facilitators’ performance in digital SGTs, different styles of group facilitation needed to be infused into both students’ and facilitators’ mind through pre-curricular training and consensus construction [11, 33]. We propose that continuously providing training courses on how to facilitate a digital SGT session for course should be a mandatory task for administrators/course designers/facilitators in order to increase small group effectiveness and team satisfaction.

An important finding in this study was that higher teamwork satisfaction predicted students’ preference for choosing digital SGT in the future (Table 6). A recent study suggested that preferences for digital education was relatively low for group learning processes compared to in-person one during the COVID-19 period, especially during prolonged digital learning [34]. We believe that this phenomenon likely resulted from the suboptimal teamwork satisfaction in under-prepared digital group learning sessions. To increase students’ engagement during digital courses and increase their preference, team dynamics can be an important key. On the other hand, students’ academic year might suggest the presence of other underlying factors influencing students’ choice (Table 6). In NTU-CM, our curricular design of SGT feature different disciplines in each academic year. SGTs for the second-year students’ focus more on medical humanity and social issues, and the SGT involving third- and fourth-year medical students was directed toward basic medical science and contained clinical cases for discussion on pathophysiological aspects. Humanity- and sociology-oriented SGT cases relied more on self-reflection and the understanding of the holistic care [35]. On the other hand, basic medical science-oriented SGT may require more knowledge for comprehension and skill application, and can be more convergent upon specific task(s) [36]. SGT in a digital setting likely offers another opportunity to facilitate singling out task(s) for collaborative work [11], creating an advantage for SGT participants, especially those of the fourth academic years in NTU-CM whose cases involving pathology and pharmacology. Another possibility would be that personal traits and the seniority as digital natives also affect SGT participants’ experiences and preferences for digital SGT. Further studies are needed to explore the influences of medical students’ characteristic on their learning preferences during SGT curricular design.

Our study provides new knowledge regarding the digital transformation process of medical education involving SGT. The impact of teamwork dynamics on teamwork satisfaction found in other educational context was clearly demonstrated in our SGT curricula [14]. We should aim to promote teamwork dynamics during digital SGT. In addition, we found that the facilitator role may decrease during digital SGT. Future tutor training for digital SGT may incorporate pre-curricular workshop and possibly tutor shadowing. Nevertheless, the homogenous cultural and educational background of our students and the specific themes and topics of our SGT curricula may limit the external validity of our results. Besides, interpretation of our data may be confounded by different topics of digital and conventional SGT and the tutors’ subject expertise. A crossover study may help reduce such confounding influences. Finally, during the COVID-19 pandemic, our tutors were trying to adapt themselves to the digital transformation process of medical education, too, thus weakening their tutor roles during SGT. This issue may resolve when tutors become more familiar with the digital meeting platforms and more adapted to the different dynamics in digital versus conventional SGT. Better support for the facilitators to adapt to the rapidly changing learning environment is needed.

## Conclusion

In conclusion, the results of this study demonstrate that team dynamics were independently correlated with teamwork satisfaction among students participating in conventional and digital SGT, while the role of facilitator support became less obvious during digital SGT. Better teamwork satisfaction was associated with an increased preference for digital SGT. These findings illuminate how we should optimize SGT course design, including strategies to enhance team dynamics and continuous education for facilitators tailored for online interaction skills.

## Data Availability

The datasets generated and/or analyzed during the current study are not publicly available due to data-sharing regulations imposed by the local institute but are available from the corresponding author on reasonable request.

## Abbreviations

ACGME: Accreditation council for graduate medical education
ANOVA: Analysis of variance
COVID-19: Coronavirus disease 2019
CVI: Content validity index
EFA: Exploratory factor analysis
I-CVI: Item level content validity index
KMO: Kaiser–meyer–olkin
NTU-CM: National Taiwan University College of Medicine
NTUH: National Taiwan University Hospital
S-CVI: Scale level content validity index
SGT: Small group tutorial
